# 驱动基因阳性肺腺癌在靶向治疗中病理类型转化的机制与治疗策略

**DOI:** 10.3779/j.issn.1009-3419.2020.102.22

**Published:** 2020-08-20

**Authors:** 同济 谢, 研 李, 镨元 邢

**Affiliations:** 100021 北京，国家癌症中心/国家肿瘤临床医学研究中心/中国医学科学院北京协和医学院肿瘤医院 National Cancer Center/National Clinical Research Center for Cancer/Cancer Hospital, Chinese Academy of Medical Sciences and Peking Union Medical College, Beijing 100021, China

**Keywords:** 肺肿瘤, 靶向治疗, 耐药, 病理类型转化, 机制, Lung neoplasms, Targeted therapy, Drug resistance, Histological transformation, Mechanism

## Abstract

驱动基因阳性的肺腺癌（adenocarcinoma, ADC）患者在接受靶向治疗一段时间后将不可避免地产生耐药。常见的耐药机制包括驱动基因的二次突变、非驱动基因的变异、病理类型转化和上皮间质转化等。其中病理类型转化包括肺ADC向小细胞肺癌（small cell lung cancer, SCLC）、鳞状细胞癌（squamous cell carcinoma, SCC）和大细胞神经内分泌癌（large cell neuroendocrine carcinoma, LCNEC）等病理类型发生转化。病理类型转化在降低患者生存质量的同时也对患者的后续治疗构成巨大的挑战，但目前对其机制的认知还不全面。本文将综述病理类型转化机制的研究成果以及治疗策略的选择。

## Abstract (en)

Patients with lung adenocarcinoma (ADC) who harbor drive gene mutation will inevitably develop drug resistance after receiving targeted therapy. The common mechanisms of drug resistance include secondary mutation of driver gene, change of non-driver gene, histological transformation and epithelial mesenchymal transformation. Histological transformation includes the transformation from lung ADC to small cell lung cancer (SCLC), squamous cell carcinoma (SCC), and large cell neuroendocrine carcinoma (LCNEC) and so on. Histological transformation not only has a negative impact on the quality of patients' life, but also poses great challenges to the follow-up treatment of patients. However the mechanism of transformation is still incomplete. This article will review the research results on the mechanism of histological transformation and the selection of treatment strategies.

与传统的化疗相比，以酪氨酸激酶抑制剂（tyrosine kinase inhibitor, TKI）为代表的小分子靶向治疗在驱动基因阳性非小细胞肺癌尤其是肺腺癌（adenocarcinoma, ADC）的治疗中取得了更好的疗效，然而患者在TKI治疗约1年后将不可避免地出现耐药现象^[1, 2]^。常见的耐药机制包括：驱动基因的二次突变，如表皮生长因子受体（epidermal growth factor receptor, *EGFR*）基因发生T790M耐药突变；非驱动基因的改变，如间质表皮转化因子（mesenchymal to epithelial transition factor, *MET*）基因扩增；上皮间质转化；病理类型转化^[3]^等。其中病理类型转化包括肺ADC向小细胞肺癌（small cell lung cancer, SCLC）、鳞状细胞癌（squamous cell carcinoma, SCC）、大细胞神经内分泌癌（large cell neuroendocrine carcinoma, LCNEC）等病理类型转化。尽管病理类型转化所引起的耐药比较罕见，但由于转化后的病理类型往往更具有侵袭性并且对TKI的反应不佳，这对患者和临床医生而言都是巨大的挑战：对患者而言，其生存质量在转化后下降；而临床医生则亟需找到有效的方法来应对这种耐药机制。虽然已经有许多文献对这一耐药机制的原理进行了研究，但目前的认知仍不全面。本文将在介绍几种常见的病理类型转化的基础上，综述转化机制的研究成果并总结发生转化后的常用临床方案以及治疗新进展。

## 1 病理类型转化机制

### 1.1 ADC转化为SCLC

#### 1.1.1 转化模型

2006年，Zakowski等^[3]^报道了1例45岁无吸烟史的女性肺ADC患者，在先后接受厄洛替尼与吉非替尼治疗后发生了耐药，再次活检提示为SCLC。尽管对于这种病理类型转化的机制并没有进行深入研究，但他们给出了两种可能的解释：①患者的肿瘤在治疗前就具有SCLC成分；②SCLC是由ADC在TKI治疗后转分化而来。Oser等^[4]^还提出了第三种解释，即SCLC可能是在TKI治疗后产生的新发肿瘤，尽管这一解释被他们立即否定。

对于第一种解释，不同的研究看法不一。Levacq等^[5]^认为由于确诊ADC时使用的标本量有限，尚不能排除SCLC在TKI治疗前就已经存在了。而Tenjin等^[6]^则在详尽检查手术标本后排除了SCLC与ADC在基线时共存的可能。Oser等^[4]^以及Niederst等^[7]^还通过反证法来排除共存的可能。他们认为如果在基线时便存在SCLC，根据SCLC的特性，肿瘤对TKI的反应应该较差，且应该较早出现耐药，而这与TKI在初期治疗有效并能维持较长一段时间后才发生耐药的临床现象不符。

对于第三种解释，即在TKI的作用下产生了新的SCLC，多数研究并不支持。在基因层面，通过比较转化前的ADC与转化后的SCLC的驱动基因改变，可以发现转化后的SCLC大多保留了之前ADC所具有的驱动基因改变（表 1）：如*EGFR* 19外显子突变^[6, 8-11]^和L858R突变^[9, 12]^、间变性淋巴瘤激酶（anaplastic lymphoma kinase, *ALK*）基因重排^[2, 5, 13, 14]^。而驱动基因突变在原发SCLC中十分罕见^[15]^，这说明SCLC很可能不是第二原发肿瘤^[2, 4]^；在细胞起源方面，尽管普遍认为SCLC起源于神经内分泌细胞而ADC起源于Ⅱ型肺泡上皮细胞^[15, 16]^，但Sutherland等^[17]^的研究提示SCLC也可以起源于Ⅱ型肺泡上皮。Sutherland等^[17]^通过腺病毒载体“Ad-Cre”使得小鼠Ⅱ型肺泡上皮的*RB1*与*Trp53*（与人类*TP53*基因同源）抑癌基因失活来诱发SCLC，虽然SCLC转化的试验结果在Park等^[18]^的研究中并未发生（Park等^[18]^认为这可能与其试验所选择的腺病毒载体“Ad-CreER”比Sutherland等^[17]^使用的“Ad-Cre”载体效率低有关），但这至少说明部分Ⅱ型肺泡上皮细胞具有发生SCLC转化的潜能；在人群特征（性别、吸烟史等）、影像学表现以及对依托泊苷联合卡铂的化疗反应率方面，Yang等^[8]^认为转化后的SCLC与原发SCLC不同。

**1:** 病理类型转化病例的部分信息（续表） Information of patients showing histological transformation (continued)

References	Age (yr)	Gender	Smoking history	Histological type (a)	*EGFR* mutation (b)	*EGFR* mutation (a)	*ALK* rearrangement (b)	*ALK* rearrangement (a)	Chemotherapy (b)	Chemotherapy (a)	TKI (b)	TKI (a)
[2]	67	F	N	SCLC	NA	NA	+	+	NVB+DDP^*^	CPT-11	Crizotinib; Alectinib	Alectinib
[5]	53	F	N	SCLC	-	-	+	+	PEM+DDP^*^	VP16+DDP; ADM+CTX+VP16	Crizotinib; Ceritinib	None
[6]	75	M	Y	SCLC	19del	19del	NA	NA	NVB+DDP	VP16+CBP	Afatinib	None
[8]	53	F	N	SCLC	19del	19del	NA	NA	NA	VP16+DDP	Erlotinib	Erlotinib
	63	F	N	SCLC	19del	-	NA	NA	NA	VP16+DDP	Gefitinib	None
	48	M	N	SCLC	19del	19del	NA	NA	NA	VP16+DDP	Erlotinib	Erlotinib
	42	M	Y	SCLC	L858R	-	NA	NA	NA	VP16+DDP	Gefitinib	None
	48	F	Y	SCLC	19del	19del	NA	NA	NA	VP16+DDP	Erlotinib	Erlotinib
	58	M	Y	SCLC	19del	T790M	NA	NA	NA	VP16+DDP	Erlotinib	Osimertinib
	60	M	N	SCLC	19del	-	NA	NA	NA	VP16+DDP	Erlotinib	None
[9]	57	F	N	SCLC	L858R	L858R	NA	NA	PTX+CBP	VP16+DDP	Gefitinib; Afatinib	None
	54	F	N	SCLC	19del	19del	NA	NA	PEM+DDP	GEM+DDP	Afatinib	None
	55	F	N	SCLC	19del	19del	NA	NA	None	VP16+DDP	Gefitinib	None
	59	F	N	SCLC	19del	19del	NA	NA	PEM+DDP	None	Gefitinib	Rociletinib
	68	F	N	SCLC	-	-	NA	NA	PEM+DDP	None	Gefitinib	None
	67	M	Y	SCLC	-	-	NA	NA	None	None	None	None
[10]	63	M	Y	SCLC	19del	19del	NA	NA	None	VP16+CBP^*^	Erlotinib	Erlotinib; Osimertinib
[11]	58	F	N	SCLC	19del	19del	NA	NA	GEM+DDP^*^	VP16+CBP	Icotinib	Icotinib
[12]	60	F	NA	SCLC	L858R	L858R	NA	NA	None	None	Gefitinib	Crizotinib; Osimertinib
[13]	43	F	NA	SCLC	NA	NA	+	+	PEM+DDP^*^	NA	Crizotinib; Erlotinib; Alectinib	NA
[14]	35	M	N	SCLC	NA	NA	+	+	None	VP16+CBP	Ceritinib; Alectinib; Lorlatinib	Alectinib
[40]	66	F	N	SCC	19exon^*^^*^	19exon^*^^*^	NA	NA	PEM+CBP	NA	Erlotinib	NA
[42]	68	M	Y	SCC	L858R	L858R; T790M	NA	NA	Tegafur+Uracil; PEM+CBP	None	Erlotinib	Osimertinib
[43]	44	F	Y	SCC	19del; T790M	19del	-	-	None	None	Afatinib	Osimertinib
[44]	79	F	N	SCC	19del	19del	NA	NA	PEM+CBP	None	None	Erlotinib
[45]	62	M	N	SCC	L858R	L858R	NA	NA	Tegafur+Uracil	PEM+DDP	Gefitinib	None
[46]	43	F	Y	SCC	L858R	L858R; S768I	-	NA	None	None	Gefitinib	Gefitinib
[48]	68	M	N	LCNEC	L858R	L858R	NA	NA	NVB+DDP; PTX+CBP^*^	VP16;Amrubicin	Gefitinib; Erlotinib	None
[49]	46	M	N	LCNEC	19del	19del	NA	NA	GEM+DDP NVB+DDP	NA	Gefitinib; Erlotinib	NA
[50]	57	M	N	LCNEC	19del	19del	NA	NA	None	VP16+DDP	Erlotinib	Erlotinib; Osimertinib
[51]	49	M	N	SCLC	L858R; T790M	NA	NA	NA	Docetaxel+DDP; PEM+DDP	VP16+CBP CPT-11+L-OHP^*^	Gefitinib; Osimertinib	Apatinib
M: male; F: female; N: without smoking history; Y: with smoking history; SCLC: small cell lung cancer; SCC: squamous cell carcinoma; LCNEC: large cell neuroendocrine carcinoma; *EGFR*: epidermal growth factor receptor; *ALK*: anaplastic lymphoma kinase; TKI: tyrosine kinase inhibitor; -: mutation/rearrangement negative; +: mutation/rearrangement postive; NA: not applicable; a: after histological transformation; b: before histological transformation; NVB: vinorelbine; DDP: cisplatin; CTP-11: irinotecan; PEM: pemetrexed; GEM: gemcitabine; CBP: carboplatin; VP16: etoposid; PTX: paclitaxel; L-OHP: oxaliplatin; ADM: adriamycin; CTX: cyclophosphamide. ^*^The patient accepted other chemotherapy besides the drug listed in this table. ^*^^*^The concrete type of mutation was not given.												

对于第二种解释，有两种理解：一是SCLC源自ADC，即线性进化；二是SCLC与ADC拥有共同的前体细胞，即分支进化。在Tatematsu等^[19]^研究的病例中有1例在转化前后的基因突变情况不支持线性进化模型。这个病例初始的ADC同时拥有*EGFR*基因的突变与扩增，而发生SCLC转化后只有*EGFR*突变被保留。Tatematsu等^[19]^认为治疗不太可能造成肿瘤细胞中的*EGFR*扩增被移除，他们进一步推测SCLC是由一个在基线时就存在的只具有*EGFR*突变的克隆在治疗的选择压力下幸存并逐步发展而来。Niederst等^[7]^的研究提出了一个分支进化的模型：在基线时存在一种前体细胞，这种细胞在TKI的作用下有可能转化为SCLC。这种分支进化模型与基线混合瘤模型的区别在于前者是单克隆来源，而后者是多克隆来源。Mooradian等^[10]^在对比多次病理标本的多个基因后构建了一个初步的进化树，但对于进化树上分支点的时间特征描述还不完善，并且由于所测基因数目较少，未发现第一次ADC标本的私有突变，故其进化树模型中SCLC克隆的分支点位于第一次ADC标本之后。Lee等^[20]^通过全基因组测序技术对4例患者转化前的ADC样本与转化后的SCLC样本进行分析以重建其克隆进化史。其中2例患者在SCLC转化之前还获得了T790M突变的耐药机制，在比较其SCLC样本与具有T790M突变ADC样本后发现二者在分享公有突变的同时还各自具有私有突变，这说明SCLC克隆与T790M突变ADC克隆具有共同祖先，而这与Mooradian等^[10]^所构造的进化树相似，但对SCLC克隆与更早的未产生T790M突变的ADC克隆是否也具有共同祖先未做进一步研究。Lee等^[20]^分析了另外2例SCLC转化为唯一耐药机制的病例的初次ADC样本与再次SCLC样本，结果仍是两种成分既有公有突变又有私有突变，即SCLC克隆与初次病理标本中的ADC克隆具有共同祖先。由于这2例患者的ADC样本取自TKI治疗之前，故SCLC克隆的前体（此时该克隆尚不具备SCLC表型）产生的时间可以追溯到TKI治疗之前。

#### 1.1.2 转化机制

目前比较合理的SCLC转化模型是分支进化模型，但是模型中还有许多细节需要继续研究。目前研究比较多的有以下3个问题：①SCLC的前体如果产生于TKI治疗之前，它是如何逃避TKI的选择压力的，又是如何获得那些SCLC私有突变的？②SCLC转化过程中涉及哪些信号通路以及这些通路是如何发挥作用的？③转化是否依赖于TKI或者*EGFR*突变状态，没有TKI的暴露或者*EGFR*野生型的ADC患者能否发生SCLC转化？

对于在TKI治疗中，SCLC前体是如何获得私有突变的问题，Lee等^[20]^认为这可能是SCLC的前体细胞在TKI的选择压力下首先进入一个低增殖或无增殖的状态，而这些细胞被称为“persisiter”；在随后的过程中载脂蛋白B mRNA编辑酶催化多肽（apolipoprotein B mRNA editing enzyme catalytic polypeptide, APOBEC）所介导的体细胞突变使得这些“persisiter”最终成为SCLC。这里对两个概念稍作解释。

关于persisiter：这一概念源于对细菌耐药的研究。Hobby等^[21]^于1942年发现99%的细菌在青霉素的作用下被破坏而仍有1%幸存。Bigger^[22]^发现尽管降低细菌浓度，抗生素仍然不能把细菌全部杀灭，而这些幸存细菌的后代可以恢复对抗生素的敏感性，因此用“persisiter”来命名这些幸存的细菌以区别于耐药菌“resister”。Balaban等^[23]^认为这些“persisiter”通过非遗传机制来降低自身增殖速度从而逃避抗生素的杀灭，并进一步按照细胞增殖动力学把它们分为Ⅰ型（不增殖型）与Ⅱ型（低增殖型）。Dawson等^[24]^认为肿瘤中也存在类似细菌中的“persisiter”，并在耐药性的产生中发挥作用。进一步对肿瘤“persisiter”的研究提示一小群肿瘤细胞在暴露于TKI后通过发生以组蛋白去甲基化、去乙酰化为代表的表观遗传学改变^[25]^以及包括胰岛素样生长因子1受体（insulin like growth factor 1 receptor, IGF-1R）介导的信号通路^[25]^、核因子κB（nuclear factor kappa B, NF-κB）通路^[26]^、表皮生长因子受体（epidermal growth factor receptor, EGFR）介导的Ras-MAPK与PI3K-Akt通路^[27]^在内的多条信号转导通路活性改变促使“persisiter”产生。这些可逆的改变使得肿瘤细胞处于低增殖或不增殖状态，因此可以逃避TKI的杀灭作用，最终经过后续的突变事件使得急性耐药阶段的“persisiter”，最终转变为慢性耐药的SCLC^[20, 25, 27, 28]^。

关于APOBEC：这一概念源自与体内脂质代谢相关的酶类APOBEC1。APOBEC1是产生载脂蛋白ApoB48所需要的一种酶，它通过修改ApoB100的mRNA以提前产生终止密码子而生成分子量小于ApoB100的ApoB48^[29]^。后来以APOBEC来命名与APOBEC1同源的分子所构成的蛋白质家族，称为APOBEC家族。这一家族包含A1、A2、A4、AID以及7种A3在内的11个成员^[30]^，它们在适应性免疫（B细胞体细胞高频突变从而产生多样性抗体^[31]^）、固有免疫（抗反转录病毒感染^[32]^）等多个方面发挥作用。APOBEC家族与乳腺癌^[33]^、卵巢癌^[34]^以及肺癌^[35]^等恶性肿瘤的发生发展也有关，并且主要是通过改变肿瘤细胞的遗传物质来完成的，其过程可以概括为：首先由APOBEC将胞嘧啶脱氨转化为尿嘧啶；进而根据细胞内不同酶对这个新产生的尿嘧啶的不同处理产生碱基类型的转换、颠换以及DNA的断裂^[33, 34, 36]^；最后这种遗传物质的改变为肿瘤在选择压力下进化提供了素材，并在选择压力的作用下产生以不同克隆为主的肿瘤^[33, 35]^，例如T790M突变所引起的对一代EGFR-TKI耐药的ADC^[20]^、病理类型转化后的SCLC^[20, 37]^等。关于病理类型转化，Offin等^[37]^通过分析*EGFR/RB1/TP53*三基因突变肺癌样本的突变频谱发现AID/APOBEC在发生SCLC转化前的ADC样本中的富集程度显著高于不发生SCLC转化的ADC样本，这说明APOBEC可能有易化SCLC转化的作用。Lee等^[20]^的研究进一步比较了含有T790M突变的ADC样本与转化后的SCLC样本的突变频谱，结果显示APOBEC在SCLC样本中更常见，这同样提示了APOBEC在ADC转化为SCLC过程中具有重要意义和研究前景。

对于SCLC转化过程中涉及的信号通路及其作用机制的问题，目前研究较多的是*TP53*与*RB1*抑癌基因通路以及Notch信号通路。这两个抑癌基因对于SCLC转化非常重要，在临床中发现许多SCLC转化前后发生*TP53*和/或*RB1*抑癌基因失活的病例^[5, 6, 10, 12, 14]^，Oser等^[4]^、Niederst等^[7]^以及Offin等^[37]^认为*RB1*和*TP53*的失活对于SCLC转化是必要不充分条件，Lee等^[20]^则进一步研究得出具有*TP53*与*RB1*双抑癌基因失活的患者发生SCLC转化的风险升高了42倍。这一重要性还在Sutherland等^[17]^通过病毒载体使小鼠Ⅱ型肺泡上皮的*Trp53*与*RB1*失活从而产生SCLC的试验结果中得到证明。Tenjin等^[6]^不仅认为SCLC转化与*TP53*、*RB1*相关通路有关，还发现Notch在SCLC转化后表达降低而ASCL1则表达升高，进而推测Notch信号通路对SCLC转化也有贡献，而这一通路在原发SCLC中被证明与SCLC的神经内分泌特征有关^[38]^。

对于转化是否依赖于TKI或者*EGFR*突变状态的问题，根据Lee等^[20]^的研究结果，SCLC克隆的前体细胞可能早在TKI治疗以前就从共同祖先中分化出来了，即SCLC前体细胞的产生并不依赖于TKI治疗。此外一些SCLC转化前从未接受过TKI治疗（接受手术治疗等）的患者也发生了SCLC转化^[9]^，这也说明SCLC转化并不依赖于TKI治疗。至于TKI在SCLC转化中除对ADC克隆提供选择压力外，是否还具有诱发SCLC克隆前体的产生或是加速SCLC表型获得的作用还需要进一步研究。而*EGFR*的突变状态是否对SCLC转化有影响，目前已经观察到无*EGFR*突变的患者也在治疗后发生了转化^[5, 9]^，Ferrer等^[39]^进一步分析*EGFR*突变对SCLC转化的影响发现*EGFR*突变组的中位SCLC转化时间（16个月）短于无*EGFR*突变组（26个月），虽然差异具有统计学意义（*P*=0.01），但不排除由于再次活检频率所引起的偏倚。因此SCLC转化虽然不依赖于*EGFR*突变，但*EGFR*突变也许与SCLC转化的速率相关。

#### 1.1.3 总结

根据上述对ADC发生SCLC转化机制的研究^[20, 25-28, 33-38]^，可以概括得出如下大致过程：初始ADC样本中存在多种亚克隆。在TKI的选择压力下大多数亚克隆死亡，少部分亚克隆通过转变为“persisiter”而低增殖或不增殖从而得以幸存，并在APOBEC介导的体细胞突变作用下积累遗传物质的改变，如*TP53*或*RB1*抑癌基因失活或Notch信号通路改变。当遗传物质的改变积累到一定程度时，该幸存亚克隆获得SCLC表型并加快增殖，最终于再次活检时表现为SCLC转化。

### 1.2 ADC转化为SCC

目前关于ADC转化为SCC的报道病例少于SCLC转化，对其机制的研究也比较少。类似于SCLC转化，Levin等^[40]^提出了SCC转化的三种可能：①在TKI治疗压力下ADC发生表型转化；②ADC与SCC在治疗前就共存；③SCC为第二原发肿瘤。Schoenfeld等^[41]^和Izumi等^[42]^在详细分析ADC样本后排除了混合SCC成分的可能。临床观察到发生SCC转化的患者也保留了与基线ADC一样的驱动基因改变（表 1），如19外显子突变^[40, 43, 44]^、L858R突变^[42, 45, 46]^，而这些突变在原发SCC中出现的概率很低^[15]^，这提示两种成分可能具有共同的祖先^[43]^，第二原发肿瘤可能性比较小^[40]^。因此，与SCLC转化类似，SCC转化目前最合理的模型也是共同起源模型。进一步比较转化前后基因突变的情况可以发现，尽管SCC保留了基线时的驱动基因突变，但在SCC转化后同样也有新突变的“出现”^[41, 42, 46]^和原有突变的“消失”^[41, 43]^。这表明SCC转化的过程可能也是分支进化，然而由于大规模比对转化前后突变的研究较少，这一猜测目前并未得到验证。值得注意的是，SCC转化在未接受TKI治疗的患者中也被发现，这提示SCC转化与SCLC转化一样不依赖于TKI的使用^[44, 47]^。

### 1.3 ADC转化为LCNEC

尽管目前报道LCNEC转化的病例较少，但类似的假说也被用于解释这一转化现象。Kogo等^[48]^以及Yanagisawa等^[49]^认为SCLC与LCNEC同属于神经内分泌肿瘤，其转化机制可能存在相似之处。通过对ADC标本进行详细检查排除基线时LCNEC混合的可能；通过转化后保留原有驱动基因突变（表 1），推断LCNEC与ADC具有共同起源^[48-50]^。

## 2 病理类型转化后的临床治疗策略

由于目前对于SCLC转化的研究较多，故以下总结的治疗策略、治疗新进展主要针对SCLC转化。

### 2.1 治疗策略

#### 2.1.1 化疗方案的选择

ADC发生SCLC转化后选择治疗SCLC常用的“依托泊苷+顺铂”^[5, 8, 9]^以及“依托泊苷+卡铂”^[6, 10, 11, 14, 51]^等方案。Ferrer等^[39]^的一项回顾性研究分析了SCLC转化后患者的生存数据，纳入Ⅲ期/ Ⅳ期非小细胞肺癌发生SCLC转化的患者61例（排除先前有肺神经内分泌肿瘤史的患者），并根据其*EGFR*突变状态分为突变组（48例）和无突变组（13例）。这些患者在发生SCLC转化后所接受的治疗主要为依托泊苷联合铂类化疗（突变组：38/48；无突变组：11/13），其生存数据显示SCLC转化后中位总生存期（overall survival, OS）在两组之间无统计学差异（突变组：9个月；无突变组：10个月；*P*=0.56）。

#### 2.1.2 TKI治疗药物的选择

患者在转化前未接受TKI治疗，但由于存在敏感的驱动基因异常，故转化后可根据驱动基因突变状态选择相应的TKI治疗^[44]^。患者在转化前已经接受过TKI治疗，转化后可根据驱动基因突变状态继续或再次使用原先的TKI^[2, 8, 10, 11, 14, 46, 50]^。Fujita等^[2]^报道了1例67岁不吸烟女性肺腺癌患者，有多处骨组织及脑转移，临床分期为T4N3M0。在接受长春瑞滨联合顺铂等方案的化疗以及放疗后达到部分缓解，后因其活检标本显示*ALK*重排阳性而先后接受克唑替尼、阿来替尼治疗。在接受阿来替尼治疗3个月后由于疾病进展而进行计算机断层扫描（computed tomography, CT）引导下穿刺活检，结果提示为小细胞癌，免疫组化仍提示*ALK*重排阳性。综合考虑驱动基因改变与转化后病理类型，患者在继续使用阿来替尼来应对*ALK*重排的同时接受原发SCLC常用化疗药物伊立替康的治疗。治疗后患者的肺部原发灶以及其他病灶均显示部分缓解。对于再次活检基因检测提示有相应靶向药物使用指征的患者，亦可以选择转化前未使用过的靶向药物进行单药或联合治疗，根据TKI的种类可以分为：EGFR-TKI^[8-10, 12, 42, 43, 50]^、MET抑制剂^[12]^、血管内皮生长因子受体TKI（vascular endothelial growth factor receptor TKI, VEGFR-TKI）^[51]^等。Liu等^[12]^报道了1例60岁IIIa期女性肺腺癌患者，其接受肺癌根治术后因不能耐受辅助化疗且具有L858R突变而接受吉非替尼治疗。约6年后疾病进展，活检提示SCLC转化，为寻求进一步治疗计划进行基因检测。检测结果显示除原有L858R突变外，肿瘤还获得了*CAV1-MET*融合，因此采用MET抑制剂克唑替尼联合三代EGFR-TKI奥希替尼治疗，疗效评估为部分缓解。

### 2.2 治疗新进展

目前对病理类型转化患者的治疗策略以应对原发该类型肿瘤的方案为主，如前所述的SCLC转化后，临床多使用治疗原发SCLC的化疗方案。Niederst等^[7]^认为原发SCLC细胞株与转化后SCLC株有着相似的药物敏感性，而结合目前针对原发SCLC的治疗新进展，我们提出合理的假设，即这些治疗原发SCLC的新进展可能有助于改善转化后SCLC患者的预后。

#### 2.2.1 针对B淋巴细胞瘤/白血病-2（B cell lymphoma/leukemia-2, Bcl-2）分子的药物

Navitoclax是一种同时针对Bcl-2分子与B淋巴细胞瘤/白血病-x_L_（B cell lymphoma/leukemia-x_L_, Bcl-x_L_）分子的抑制剂，但Navitoclax在IIa期临床试验中的结果却不理想。Rudin等^[52]^的研究显示在接受Navitoclax单药治疗的39例复发性SCLC患者中，总缓解率仅2.6%（1例患者达到部分缓解，无完全缓解病例），中位无进展生存期（progression-free survival, PFS）为1.5个月，中位OS为3.2个月。经分析认为降低Navitoclax疗效的因素之一是血小板减少这一不良反应的发生限制了Navitoclax抗瘤效应^[53]^。Lochmann等^[53]^的细胞及动物试验探究了选择性Bcl-2抑制剂Venetoclax的抗肿瘤活性，结果显示Venetoclax在Bcl-2高表达的SCLC中效果良好，且由于不作用于Bcl-x_L_而避免了血小板减少的发生。Lakhani等^[54]^对Bcl-2分子与Bcl-x_L_分子的双靶点新药APG-1252治疗进展期SCLC的Ⅰ期临床试验（NCT: 03080311）研究结果显示在剂量爬升过程中尚未出现血液学不良反应，其疗效与安全性仍在进一步研究中。尽管上述三项研究针对的不是转化后SCLC，但其研究结果为治疗转化后SCLC提供了新思路。例如Niederst等^[7]^的研究显示转化后SCLC细胞株对Navitoclax表现出良好的敏感性，这提示Navitoclax用于治疗转化后SCLC可能有一定的价值，但考虑到其单药治疗在原发SCLC患者中引起的血小板减少^[52]^，可以在未来的试验中探究含Navitoclax的多药联合治疗，或是将血小板减少风险低的新药Venetoclax^[53]^、APG-1252^[54]^等用于转化后SCLC的治疗。

#### 2.2.2 针对δ样蛋白3（delta-like protein 3, DLL-3）分子的药物

目前研究较多的靶向DLL-3分子的药物是一种抗体偶联药物Rovalpituzumab Tesirine（Rova-T）。一项Rova-T治疗复发性SCLC的Ⅰ期临床研究结果^[55]^显示，接受有活性剂量Rova-T治疗的26例高表达DLL-3（被定义为表达DLL-3的肿瘤细胞≥50%）患者的客观缓解率（objective response rate, ORR）为38%（10/26），疾病控制率为88%（23/26），中位PFS为4.3个月；而另一项Rova-T治疗复发性SCLC的Ⅱ期临床试验结果^[56]^却显示，接受0.3 mg/kg Rova-T治疗的238例高表达DLL-3（被定义为表达DLL-3的肿瘤细胞≥75%）患者的ORR为14.3%（34/238），中位PFS为3.8个月，中位OS为5.7个月，没有能够重复Ⅰ期临床试验的良好结果。除此之外，一项Rova-T的Ⅲ期临床试验MERU（NCT: 03033511）由于Rova-T相对安慰剂没能改善患者生存，故AbbVie已宣布中止MERU研究。尽管上述针对原发SCLC患者临床试验结果并不理想，但是基于Ⅰ期临床试验令人鼓舞的结果，Rova-T将来是否能够应用于转化后SCLC仍值得探讨。

#### 2.2.3 针对程序性死亡受体1（programmed cell death protein 1, PD-1）分子与程序性死亡配体1（programmed cell death ligand 1, PD-L1）分子的药物

目前免疫检查点抑制剂应用于SCLC复发或初治患者已经取得了不错的疗效，根据CheckMate 032的研究结果，Nivolumab单药治疗复发性SCLC患者的ORR为11.9%，中位PFS为1.4个月，中位OS为5.6个月^[57]^；根据IMpower 133的研究结果，Atezolizumab联合化疗治疗SCLC初治患者的ORR为60.2%，中位PFS为5.2个月，中位OS为12.3个月^[58]^；CASPIAN的研究结果显示，Durvalumab联合化疗治疗SCLC初治患者的ORR为68%，中位PFS为5.1个月，中位OS为13.0个月^[59]^。上述试验结果提示免疫检查点抑制剂用于转化后SCLC具有巨大的潜力。

## 3 总结

驱动基因阳性肺ADC患者在TKI治疗过程中发生病理类型转化是一种非常复杂的耐药机制，目前最合理的假说是具有共同起源细胞的分支进化模型。转化后的治疗方法仍以化疗和TKI治疗为主，但治疗原发SCLC的新进展为应对转化后SCLC提供了新思路，这为进一步改善驱动基因阳性ADC患者的预后提供了可能。

## References

[1] Sequist LV, Waltman BA, Dias-Santagata D (2011). Genotypic and histological evolution of lung cancers acquiring resistance to EGFR inhibitors. Scie Transl Med.

[2] Fujita S, Masago K, Katakami N (2016). Transformation to SCLC after treatment with the ALK inhibitor alectinib. J Thorac Oncol.

[3] Zakowski MF, Ladanyi M, Kris MG (2006). *EGFR* mutations in small-cell lung cancers in patients who have never smoked. N Engl J Med.

[4] Oser MG, Niederst MJ, Sequist LV (2015). Transformation from non-small-cell lung cancer to small-cell lung cancer: molecular drivers and cells of origin. Lancet Oncol.

[5] Levacq D, D'Haene N, de Wind R (2016). Histological transformation of *ALK* rearranged adenocarcinoma into small cell lung cancer: A new mechanism of resistance to ALK inhibitors. Lung Cancer.

[6] Tenjin Y, Nakamura K, Ishizuka S (2019). Small cell lung cancer derived from adenocarcinoma with mutant epidermal growth factor receptor provides a signature of transcriptional alteration in tumor cells. Intern Med.

[7] Niederst MJ, Sequist LV, Poirier JT (2015). RB loss in resistant *EGFR* mutant lung adenocarcinomas that transform to small-cell lung cancer. Nat Commun.

[8] Yang H, Liu L, Zhou C (2019). The clinicopathologic of pulmonary adenocarcinoma transformation to small cell lung cancer. Medicine (Baltimore).

[9] Ahn S, Hwang SH, Han J (2016). Transformation to small cell lung cancer of pulmonary adenocarcinoma: clinicopathologic analysis of six cases. J Pathol Transl Med.

[10] Mooradian MJ, Piotrowska Z, Drapkin BJ (2017). Clonal evolution and the role of serial liquid biopsies in a case of small-cell lung cancer-transformed *EGFR* mutant non-small-cell lung cancer. JCO Precis Oncol.

[11] Han C, Shuyan Meng, Lv M (2019). Small-cell lung cancer transformation in a pulmonary adenocarcinoma patient as an acquired resistance mechanism to icotinib: a case report and review. Int J Clin Exp Med.

[12] Liu J, Li X, Peng J (2019). A novel *CAV1-MET* fusion in SCLC transformation responds to crizotinib and osimertinib treatment. J Thorac Oncol.

[13] Takegawa N, Hayashi H, Iizuka N (2016). Transformation of *ALK* rearrangement-positive adenocarcinoma to small cell lung cancer in association with acquired resistance to alectinib. Ann Oncol.

[14] Ou SI, Lee TK, Young L (2017). Dual occurrence of *ALK* G1202R solvent front mutation and small cell lung cancer transformation as resistance mechanisms to second generation ALK inhibitors without prior exposure to crizotinib. Pitfall of solely relying on liquid re-biopsy?. Lung Cancer.

[15] Swanton C, Govindan R (2016). Clinical implications of genomic discoveries in lung cancer. N Engl J Med.

[16] Ouadah Y, Rojas ER, Riordan DP (2019). Rare pulmonary neuroendocrine cells are stem cells regulated by Rb, p53, and Notch. Cell.

[17] Sutherland KD, Proost N, Brouns I (2011). Cell of origin of small cell lung cancer: inactivation of Trp53 and Rb1 in distinct cell types of adult mouse lung. Cancer Cell.

[18] Park KS, Liang MC, Raiser DM (2011). Characterization of the cell of origin for small cell lung cancer. Cell Cycle.

[19] Tatematsu A, Shimizu J, Murakami Y (2008). Epidermal growth factor receptor mutations in small cell lung cancer. Clin Cancer Res.

[20] Lee JK, Lee J, Kim S (2017). Clonal history and genetic predictors of transformation into small-cell carcinomas from lung adenocarcinomas. J Clin Oncol.

[21] Hobby GL, Meyer K, Chaffee E (1942). Observations on the mechanism of action of penicillin. Exp Biol Med.

[22] Bigger JW (1944). Treatment of staphylococcal infections with penicillin. Lancet.

[23] Balaban NQ, Merrin J, Chait R (2004). Bacterial persistence as a phenotypic switch. Science.

[24] Dawson CC, Intapa C, Jabra-Rizk MA (2011). "Persisters": survival at the cellular level. PLoS Pathog.

[25] Sharma SV, Lee DY, Li B (2010). A Chromatin-mediated reversible drug-tolerant state in cancer cell subpopulations. Cell.

[26] Lantermann AB, Chen D, McCutcheon K (2015). Inhibition of casein kinase 1 alpha prevents acquired drug resistance to erlotinib in *EGFR*-mutant non-small cell lung cancer. Cancer Res.

[27] Tetsu O, Phuchareon J, Eisele DW (2015). AKT inactivation causes persistent drug tolerance to EGFR inhibitors. Pharmacol Res.

[28] Ramirez M, Rajaram S, Steininger RJ (2016). Diverse drug-resistance mechanisms can emerge from drug-tolerant cancer persister cells. Nat Commun.

[29] Powell LM, Wallis SC, Pease RJ (1987). A novel form of tissue-specific RNA processing produces apolipoprotein-B48 in intestine. Cell.

[30] Burns MB, Leonard B, Harris RS (2015). APOBEC3B: Pathological consequences of an innate immune DNA mutator. Biomed J.

[31] Di Noia JM, Neuberger MS (2007). Molecular mechanisms of antibody somatic hypermutation. Annu Rev Biochem.

[32] Desimmie BA, Delviks-Frankenberrry KA, Burdick R (2014). Multiple APOBEC3 restriction factors for HIV-1 and one vif to rule them all. J Mol Biol.

[33] Harris RS (2015). Molecular mechanism and clinical impact of *APOBEC3B*-catalyzed mutagenesis in breast cancer. Breast Cancer Res.

[34] Leonard B, Hart SN, Burns MB (2013). *APOBEC3B* upregulation and genomic mutation patterns in serous ovarian carcinoma. Cancer Res.

[35] Jamal-Hanjani M, Wilson GA, McGranahan N (2017). Tracking the evolution of non-small-cell lung cancer. N Engl J Med.

[36] Henderson S, Fenton T (2015). *APOBEC3* genes: retroviral restriction factors to cancer drivers. Trends Mol Med.

[37] Offin M, Chan JM, Tenet M (2019). Concurrent *RB1* and *TP53* alterations define a subset of *EGFR*-mutant lung cancers at risk for histologic transformation and inferior clinical outcomes. J Thorac Oncol.

[38] George J, Lim JS, Jang SJ (2015). Comprehensive genomic profiles of small cell lung cancer. Nature.

[39] Ferrer L, Giaj Levra M, Brevet M (2019). A brief report of transformation from NSCLC to SCLC: Molecular and therapeutic characteristics. J Thorac Oncol.

[40] Levin PA, Mayer M, Hoskin S (2015). Histologic transformation from adenocarcinoma to squamous cell carcinoma as a mechanism of resistance to *EGFR* inhibition. J Thorac Oncol.

[b41] 41Schoenfeld AJ, Chan JM, Kubota D, *et al.* Tumor analyses reveal squamous transformation and off-target alterations as early resistance mechanisms to first-line osimertinib in *EGFR*-mutant lung cancer. Clin Cancer Res, 2020. pii: clincanres.3563.2019. doi: 10.1158/1078-0432.CCR-19-3563

[42] Izumi H, Yamasaki A, Ueda Y (2018). Squamous cell carcinoma transformation from *EGFR*-mutated lung adenocarcinoma: A case report and literature review. Clin Lung Cancer.

[43] Bruno R, Proietti A, Ali G (2017). Squamous cell transformation and *EGFR* T790M mutation as acquired resistance mechanisms in a patient with lung adenocarcinoma treated with a tyrosine kinase inhibitor: A case report. Oncol Lett.

[44] Le T, Sailors J, Oliver DH (2017). Histologic transformation of *EGFR* mutant lung adenocarcinoma without exposure to EGFR inhibition. Lung Cancer.

[45] Shinohara S, Ichiki Y, Fukuichi Y (2018). Squamous cell carcinoma transformation from adenocarcinoma as an acquired resistance after the EGFR TKI therapy in (*EGFR*-mutated) non-small cell lung cancer. J Thorac Dis.

[46] Longo L, Mengoli MC, Bertolini F (2017). Synchronous occurrence of squamous-cell carcinoma "transformation" and *EGFR* exon 20 S768I mutation as a novel mechanism of resistance in *EGFR*-mutated lung adenocarcinoma. Lung Cancer.

[47] Hsu CL, Chen KY, Kuo SW (2017). Histologic transformation in a patient with lung cancer treated with chemotherapy and pembrolizumab. J Thorac Oncol.

[48] Kogo M, Shimizu R, Uehara K (2015). Transformation to large cell neuroendocrine carcinoma as acquired resistance mechanism of EGFR tyrosine kinase inhibitor. Lung Cancer.

[49] Yanagisawa S, Morikawa N, Kimura Y (2012). Large-cell neuroendocrine carcinoma with epidermal growth factor receptor mutation: possible transformation of lung adenocarcinoma. Respirology.

[50] Baglivo S, Ludovini V, Sidoni A (2017). Large cell neuroendocrine carcinoma transformation and *EGFR*-T790M mutation as coexisting mechanisms of acquired resistance to EGFR-TKIs in lung cancer. Mayo Clin Proc.

[51] Chen S, He Y, Liu J (2019). Third-generation TKI resistance due to SCLC transformation: A case report and brief review. OncoTargets Ther.

[52] Rudin CM, Hann CL, Garon EB (2012). Phase Ⅱ study of single-agent navitoclax (ABT-263) and biomarker correlates in patients with relapsed small cell lung cancer. Clin Cancer Res.

[53] Lochmann TL, Floros KV, Naseri M (2018). Venetoclax is effective in small cell lung cancers with high BCL-2 expression. Clin Cancer Res.

[54] Lakhani NJ, Rasco DW, Tolcher AW (2018). A phase Ⅰ study of novel dual Bcl-2/Bcl-xL inhibitor APG-1252 in patients with advanced small cell lung cancer (SCLC) or other solid tumor. J Clin Oncol.

[55] Rudin CM, Pietanza MC, Bauer TM (2017). Rovalpituzumab tesirine, a DLL3-targeted antibody-drug conjugate, in recurrent small-cell lung cancer: a first-in-human, first-in-class, open-label, phase 1 study. Lancet Oncol.

[56] Morgensztern D, Besse B, Greillier L (2019). Efficacy and safety of rovalpituzumab tesirine in third-line and beyond patients with DLL3-expressing, relapsed/refractory small-cell lung cancer: Results from the phase Ⅱ TRINITY study. Clin Cancer Res.

[57] Ready N, Farago AF, Braud FD (2018). Third-line nivolumab monotherapy in recurrent SCLC: CheckMate 032. J Thorac Oncol.

[58] Horn L, Mansfield AS, Szczęsna A (2018). First-line atezolizumab plus chemotherapy in extensive-stage small-cell lung cancer. N Engl J Med.

[59] Paz-Ares L, Dvorkin M, Chen Y (2019). Durvalumab plus platinum-etoposide versus platinum-etoposide in first-line treatment of extensive-stage small-cell lung cancer (CASPIAN): a randomised, controlled, open-label, phase 3 trial. Lancet.

